# High School Composition and Health Outcomes in Adulthood: A Cohort Study

**DOI:** 10.3390/ijerph18073799

**Published:** 2021-04-06

**Authors:** Alison K. Cohen, Emily J. Ozer, David H. Rehkopf, Barbara Abrams

**Affiliations:** 1 Department of Epidemiology & Biostatistics, School of Medicine, University of California, San Francisco, CA 94158, USA; 2 School of Public Health, University of California Berkeley, Berkeley, CA 94720, USA; eozer@berkeley.edu (E.J.O.); babrams@berkeley.edu (B.A.); 3 Departments of Epidemiology & Population Health and Medicine, School of Medicine, Stanford University, Stanford, CA 94305, USA; drehkopf@stanford.edu

**Keywords:** USA, educational status, obesity, self-rated health, social determinants of health, social epidemiology, school composition

## Abstract

Background: A multitude of empirical evidence documents links between education and health, but this focuses primarily on educational attainment and not on characteristics of the school setting. Little is known about the extent to which aggregate characteristics of the school setting, such as student body demographics, are associated with adult health outcomes. Methods: We use the U.S. nationally representative National Longitudinal Survey of Youth 1979 cohort to statistically assess the association between two different measures of high school student composition (socioeconomic composition, racial/ethnic composition) and two different health outcomes at age 40 (self-rated health and obesity). Results: After adjusting for confounders, high school socioeconomic composition, but not racial/ethnic composition, was weakly associated with both obesity and worse self-rated health at age 40. However, after adding adult educational attainment to the model, only the association between high school socioeconomic composition and obesity remained statistically significant. Conclusions: Future research should explore possible mechanisms and also if findings are similar across other populations and in other school contexts. These results suggest that education policies that seek to break the link between socioeconomic composition and negative outcomes remain important but may have few spillover effects onto health.

## 1. Introduction

**Figure 1:**
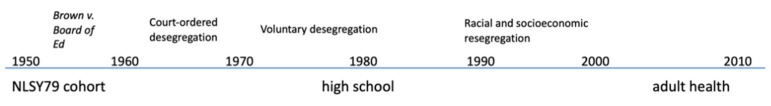
Educational historical context as related to the present study.

Myriad researchers have identified education as a key social determinant of health. The empirical research to support this claim focuses almost entirely on the positive association between educational attainment and health [1,2,3,4]. Education, however, is comprised of not only the amount attained (quantity) but also the quality of the education, including characteristics of the school setting. Reviews of research on education and health suggest that the quality of the school experience, not just the quantity, may affect health [5,6]. In recent decades, education researchers and practitioners have focused on policies to improve the school setting. For example, there is a long history of education policies focusing on school composition—primarily racial/ethnic, but also socioeconomic—with the goal of positive life outcomes for students. Little is known, however, about the extent to which such school setting characteristics, especially in the decades following court-mandated desegregation, are associated with health outcomes in adulthood (Figure 1). This paper investigates the relationship between the composition of the high school student body and health outcomes at age 40 in a recent, nationally representative American cohort.

### 1.1. School Composition

Social epidemiologists have studied neighborhoods and health, yielding a rich literature investigating compositional effects or the extent to which aggregate characteristics of neighborhood residents may be associated with health outcomes among individuals living in those neighborhoods [6,7,8]. Consistent with this social epidemiologic literature, the present study approaches the question of school compositional effects in the same way. Specifically, we consider to what extent high schools’ racial/ethnic composition and socioeconomic composition may be associated with health outcomes in adulthood.

We first provide a conceptual and historical context for investigating these educational exposures. Over the years, the racial/ethnic make-up of schools has been a major concern of education policies and debates [9], from segregation to desegregation and integration policies [10,11,12]. However, in more recent decades, the desegregation policies unraveled, and schools have become more demographically homogenous [13]. This has been driven by de facto residential shifts that create more racially/ethnically and socioeconomically homogenous neighborhoods and/or school districts [14,15] and courts opting away from continued enforcement of desegregation policies [16]. 

While the majority of school composition literature and policy may be focused on race/ethnicity, socioeconomic composition is also important and is associated with educational outcomes [17]. The percent of students eligible for free or reduced-price lunch is often used as an indicator of socioeconomic disadvantage, defined broadly [18]. Under the National School Lunch Program, students become eligible for free or reduced-price lunch either by income (less than 185% of federal poverty level) or by participation in other programs (including having a foster child present in the home or participating in federal financial assistance programs that have their own income eligibility criteria, such as Temporary Assistance for Needy Families (TANF)) [18]. A major education policy, Title I of the Elementary and Secondary Education Act (ESEA), acknowledges that socioeconomically disadvantaged students require more educational resources and identifies “Title I schools” (schools where ≥40% of students are eligible for free lunch) to receive additional, compensatory resources [19].

These two school composition characteristics have been affected by historical trends and are meaningful for educational outcomes. This paper builds upon that foundation and focuses on any potential associations between those variables and health.

### 1.2. Social Patterning of Health 

Generally, the life course literature suggests that social settings in childhood and adolescence are relevant for health later in life [20]. High school student composition is one such social setting. An inductive case for a link between high school composition and adult health could include mechanisms such as school climate, peer effects, and economic opportunities.

#### 1.2.1. Conceptualizing and Assessing Health

Health can be framed as a global construct related to quality of life that can also be operationalized via anthropometric measurements of specific health characteristics. When considering the social patterning of health outcomes, it can be useful to see if similar trends emerge across different health outcomes, as would be suggested per the fundamental causes of disease theory [21]. For the purposes of this paper, we focus on two: self-rated health, a global measure of health, and obesity, an anthropometric measure of a specific facet of health. In addition to being meaningful health outcomes themselves, they are also clinically significant harbingers of other adverse health outcomes. For example, both self-rated health [22,23,24] and obesity [25,26] are strongly associated with morbidity and mortality. 

#### 1.2.2. Empirical Data

It has also been well documented that there is a social patterning of both self-rated health and obesity, such that those with more education report better self-rated health [27] and are less likely to be obese [28]. Race/ethnicity may modify the association between educational attainment and both self-rated health [29] and obesity [30]. When race/ethnicity modifies the association between educational attainment and other health variables, that may reflect differences in educational quality [5], an alternate explanation is that education leads to more social mobility and opportunity for people of some races/ethnicities, for both men [31] and women [32]. This paper explores the potential role of educational quality. 

We identified few studies that examined the relationship between measures of the educational experience and adult self-rated health. In one quasi-experimental study of children born in the 1950s–1970s who lived in U.S. communities that had segregated schools, court-ordered desegregation was associated with improved school quality for black students, which was in turn associated with self-rated health in adulthood. For white students, no change in school quality or health outcomes was observed [33]. Among children born in the 1950s in Scotland, those who attended primary schools where a higher proportion of students’ fathers had professional and managerial jobs reported better self-rated health approximately forty years later [34]. More recently, in the U.S. National Longitudinal Study of Adolescent Health (Add Health), where students attended high school in the mid-1990s, school socioeconomic composition was associated with self-rated health, and the association between school racial/ethnic composition and self-rated health varied by race/ethnicity [35]. Also in Add Health, attending a school with more white students was associated with worse self-rated health for black students, better self-rated health for white students, and there was no association for Latinx students [36]. Since only one study has focused on adult obesity as an outcome, we also found that school racial composition was associated with body mass index (BMI) cross-sectionally among adolescent girls [37] and that school socioeconomic composition in adolescence was associated with BMI in young adulthood [38]. 

A handful of other studies have considered how school composition may relate to other health outcomes in U.S. samples. One study found a cross-sectional association between physical activity and both school-level median household income and racial/ethnic composition in Add Health adolescents [39]. Among Add Health participants, school racial composition was associated with depressive symptoms in adolescence cross-sectionally [40] and also into early adulthood [41] for black people, and school socioeconomic composition was associated with physiological dysregulation in young adulthood [42]. In a different cohort of older African American adults born in the 1930s–1950, attending desegregated (as opposed to segregated) schools was associated with lower sense of control and poorer physical performance [43]. Taken together, the current small body of research suggests that it is worth continuing to explore any potential associations between school composition and health, especially in a national sample that can control for childhood socioeconomic position. 

#### 1.2.3. Interaction between Individual and School Characteristics

It is also worth considering if individual demographic characteristics interact with school-level demographic characteristics to affect health outcomes. For example, individual race/ethnicity or socioeconomic position may modify the association between these high school student body composition characteristics and adult health outcomes. While some have found no difference in the relationship between school composition and student educational outcomes by individual socioeconomic status [44], others have found that both individual and group-level socioeconomic position are associated with health outcomes [45], and so it is possible that these different levels could interact. 

### 1.3. Rationale for the Present Study

Here, we investigate the association between high school student racial/ethnic and socioeconomic composition with their self-rated health and obesity at age 40. We also investigate the extent to which these associations are modified by individual-level race/ethnicity and socioeconomic position. As described earlier, the school composition variables were chosen for their relevance in education history and policy, and the two health variables are two conceptually different, clinically relevant, socially patterned constructs. 

## 2. Methods

### 2.1. Participants

The National Longitudinal Survey of Youth 1979 cohort (NLSY) is a prospective longitudinal cohort study conducted by the USA’s Bureau of Labor Statistics [46]. Using a complex multistage sampling approach, it recruited 14–21-year-olds in 1979 from randomly sampled households and has followed them prospectively since. This manuscript uses data collected through when participants reached age 40 (through 2010). Seventy-six percent of the original cohort were alive, eligible, and continuing to participate in 2010; excluding those who were deceased, the proportion retained was 80.6% [47]. Some demographic groups of interest were oversampled; with weighting, the study was designed to be nationally representative [46]. Additionally, the administrators of the high schools that NLSY participants attended were invited to complete a survey reporting information about their school; we only included NLSY participants who also had these administrator data for the purposes of this study. The data used were de-identified and publicly available over the Internet; thus the University of California Berkeley Committee for the Protection of Human Subjects deemed these analyses exempt from review. 

The sample size for calculating each of the odds ratios varied due to missing data; the smallest sample size used for the purposes of calculating an odds ratio was 3704 (for model 3 for percent disadvantaged and obesity). This is 49.0% of those who were still participating in 2010 (*n* = 7565), and 29.2% of the original sample (*n* = 12,686). This level of attrition is relatively common for comparable longitudinal cohort studies [32], and research suggests that the attrition in the NLSY would only bias estimates of associations between social factors and health towards the null [48]. 

### 2.2. Instrumentation

#### 2.2.1. School Composition

School administrators reported high school characteristics. We identified two composition characteristics for this analysis: the percent of students classified as economically disadvantaged per National School Lunch Program guidelines and the percent of students who were White (given that the distribution of minority groups differs across the United States, this was the most consistent way to consider the presence of minority students nationwide). 

#### 2.2.2. Health Outcomes

Individuals self-reported their health at age 40 or 41 on a 5-point Likert scale as excellent, very good, good, fair, or poor. Although self-rated health is a very generic question, it has been found to be a very strong predictor of future morbidity and mortality [23,25] and is interpreted consistently across multiple subgroups [49,50]. We chose to operationalize it in its original form, as an ordered categorical variable, rather than create a binary version of this variable. 

Obesity was a binary variable, calculated as age 40–41 BMI ≥ 30, where BMI = weight in kilograms/(height in meters, squared) [51]. Obesity is often used instead of BMI as a continuous measure because it has greater clinical implications for morbidity [52] and mortality [26]. In the NLSY, BMI was calculated from regression-calibrated self-reported weight and height based on US National Health and Nutrition Examination Survey data of measured and recalled weight and height [53,54].

### 2.3. Data Analysis

We calculated odds ratios using logistic regression (obesity) or ordered logistic regression (self-rated health) in Stata 11.2, accounting for sampling design with survey weights.

We ran three models for each health outcome. The first model reported the results from a bivariate regression analysis (adjusted for no covariates). The second model adjusted for potential confounding variables: maternal and paternal educational attainment (as the most directly relevant measure of family socioeconomic position), childhood residential geography (urbanicity, growing up in the South), race/ethnicity (measured in the NLSY as black, Hispanic, non-black non-Hispanic white), gender, birth year, and if the individual spoke a foreign language at home as a child. We did not adjust for adolescent health outcomes, as these were plausibly along the causal pathway rather than in advance of the exposure of interest. The third model adjusted for the same variables as model 2 and also adjusted for educational attainment at age 25 as a possible mediator [55]. 

We also tested for interaction by two individual-level variables separately: race/ethnicity and socioeconomic position (as measured by maternal educational attainment, as a categorical variable of less than high school graduate, high school graduate but not college graduate, and college graduate or more). We added interaction terms to new models that adjusted for all of the variables in model 3. Since interaction analyses are typically underpowered [56], we used an a priori *p*-value cutpoint of *p* < 0.1 to assess significance. 

## 3. Results

### 3.1. Overall Population

Out of the 7961 respondents with information about both high school characteristics and self-rated health, 21.6% rated their health as excellent, 37.3% as very good, 27.9% as good, 11.0% as fair, and 2.2% as poor. In general, both those who were obese at age 40 and those with poorer health went to high schools with less advantaged student bodies, had less highly educated mothers and fathers, and had lower educational attainment themselves at age 25. More information about the distribution of characteristics is available in Table 1.

### 3.2. Self-Rated Health

All of the ordered odds ratios (ORs) for associations between measures of high school student composition and self-rated health were quite close to the null (Table 2). While all of the bivariate ordered odds ratios (model 1) were statistically significant, after adjusting for confounders (model 2), no associations remained statistically significant. For all three school composition variables, the point estimates moved slightly closer to the null from model 1 to model 2, and the confidence intervals for model 1 and model 2 were mutually exclusive, suggesting that the covariates included in model 2 confounded the associations of interest. In Model 3, after accounting for educational attainment at age 25, none of the ORs were significant.

Although there were no significant main effects after adjusting for childhood socioeconomic position and demographic confounders, we still tested for the interaction terms we had hypothesized a priori, since null main effects can mask significant associations in certain demographic subgroups. Interaction results were mixed. 

For the association between the percent of students who were disadvantaged and self-rated health, there was a significant interaction by race/ethnicity (*p* = 0.08 for the set of interaction terms), implying that the association varied by race/ethnicity. However, each race/ethnicity-specific ordered OR was null (ordered OR for non-black non-Hispanic participants: 0.98 (95% CI: 0.96, 1.01); ordered OR for non-Hispanic black participants: 1.02 (95% CI: 0.99, 1.04); ordered OR for Hispanic participants: 1.02 (95% CI: 0.98, 1.06)). The student disadvantage- self-rated health association did not appear to vary by maternal education (*p* = 0.67). 

For the relationship between student racial/ethnic composition and self-rated health, there was no significant interaction by individual race/ethnicity (*p* = 0.51) or maternal education (*p* = 0.22). 

### 3.3. Obesity

From model 1 to model 2, the point estimates all moved closer to the null, suggesting that it is necessary to adjust for the covariates included in model 2 since they appeared to confound the associations. After adjusting for potential confounders (model 2), no associations remained significant; the same was true after adding educational attainment at age 25 to the model (model 3) (Table 3). 

Despite no statistically significant main effects, we still investigated possible effect measure modification by individual-level characteristics. Neither race/ethnicity (*p* = 0.43) nor maternal education (*p* = 0.66) modified the association between percent of disadvantaged students in the high school and obesity at age 40. Further, there was no interaction by race/ethnicity for racial/ethnic student composition (*p* = 0.42), but there was an interaction by maternal education (*p* = 0.03). Results from this interaction analysis suggested that among those whose mothers had graduated from college or beyond, a five-percentage-point increase in the proportion of the high school student body who were white was inversely associated with obesity at age 40 (OR: 0.97, 95% CI: 0.94, 1.00 (*p* = 0.04)). There was no association for individuals whose mothers graduated from high school but not college (OR: 1.00, 95% CI: 0.98, 1.02) or whose mothers did not graduate from high school (OR: 1.00, 95% CI: 0.98, 1.02). 

## 4. Discussion

While a large body of literature has documented associations between high school student composition and educational outcomes, there does not appear to be substantial spillover effects of high school composition for adult health, especially after controlling for the rich set of confounding variables in this U.S. national longitudinal cohort (NLSY79). The purpose of controlling for so many individual-level demographic and socioeconomic variables was to rigorously assess the relationship between high school composition and adult health in a nationally representative sample of Americans. In alignment with some of the previous studies of school composition and adult health mentioned [33,57] but not others [34,35,41,43], all of the odds ratios for the associations between high school composition variables (racial/ethnic composition, socioeconomic composition) and health (self-rated health, obesity) were null or quite close to the null. 

This study responds to calls by social epidemiologists studying neighborhoods and health to also consider how the school setting may relate to health [58], as well as those by life course epidemiologists suggesting the importance of adolescent experiences for health outcomes later in life [59], as well as those by social epidemiologists to better understand education as an exposure [60]. Our findings suggest that any direct associations between high school student composition and health do not persist or persist only weakly into middle age. This is counter to findings that court-ordered racial desegregation had beneficial health outcomes (albeit only for black people) in the United States [33] and that school socioeconomic composition was associated with health outcomes in a Scottish sample [34], but aligned with other, cross-sectional studies that have found no association between school composition measures and self-rated health in Swedish adolescents [61]. Nevertheless, there are reasons other than health benefits why school systems should work towards student racial/ethnic and socioeconomic diversity in schools. 

A major strength of this study is the analysis of a diverse, nationally representative cohort with over two decades of follow-up. Additionally, we were able to control for a rich set of socioeconomic variables from across the life course, to help isolate the role of school composition. We also had strong measures of school composition, because they were based on direct school administrator reports, rather than study participant recall or proxy measures like neighborhood composition. 

However, there are also limitations. Obesity was based on self-reported height and weight; to help address a possible reporting bias, we used regression calibration to account for known ways in which Americans systematically misreport these measures. We also acknowledge that free and reduced-price lunch is an increasingly limited measure of socioeconomic position [19]. School composition could affect student outcomes through many possible pathways. We encourage future researchers to explore possible mechanisms more comprehensively and with diverse analytic techniques. Further, our analyses could have been biased in different ways. Given that researchers suggest that attrition in the NLSY may bias estimates of associations between social factors and health towards the null [48], it is possible that there is a true association between high school student composition and adult health that our study failed to detect, but given the precision of our estimates of a null association, this seems less likely. 

We also only had information about the high school student body and not their elementary and/or middle school experiences. Future researchers could assess the extent to which the composition of participants’ elementary, middle, and high schools may have different implications for health. Similarly, we only had school-level composition variables, when classroom composition may also be related to adult outcomes in more nuanced ways. For example, schools that are diverse at the school level may have tracking such that students go through school in classes that are much more homogeneous [62], and this within-school segregation may have implications for health [63]. 

Additionally, our observational data limited our ability to make causal inferences. While natural experiments exist that assessed the health effects of desegregation through quasi-random timing of court decisions [33], the trend of resegregation has happened more perniciously and less systematically over time, making it more difficult to isolate possible natural experiments. We encourage future researchers to identify creative natural experiments in the current context to further explore this question. 

## 5. Conclusions

Results were relatively consistent across both obesity and self-rated health, suggesting that this may be illustrative of the relationship more generally between school composition and health. For both health outcomes, the studied associations were weak, and no main effects were statistically significant after adjusting for confounding. For both self-rated health and obesity, we observed some interactions between individual-level variables and school composition, although they were not consistent across health outcomes. Our interaction findings for self-rated health were unexpected. The literature suggests that historically marginalized students, like students of color, would be more negatively affected by negative school characteristics because they have fewer resources to counteract these negative effects in their home and community environments [16]. However, we found that the proportion of students who dropped out from a participant’s school was associated with improved self-rated health in adulthood for black people and Hispanic/Latinx people only. This finding merits further inquiry and replication in other datasets to determine if this was due to chance or provides new insights regarding the complexity of the effects of cumulative disadvantage. 

Education policymakers have already identified economic disadvantage as an issue for educational outcomes and developed policies like Title I and free and reduced-priced meals to attempt to target this population. The findings of the present study suggest that high school composition may play a small role in the link between schooling and health outcomes that persist into adulthood, but that this question requires further investigation in other national cohorts. 

## Figures and Tables

**Table 1:** Descriptive statistics, presented for the total population, by self-rated health status and obesity status.

	Non-Obese at Age 40 (*n* = 2764)	Obese at Age 40 (*n* = 1465)	Excellent/Very Good Self-Rated Health (*n* = 2870)	Good/Fair/ Poor Self-Rated Health (*n* = 1882)	Total Population (*n* = 4224)
Proportion of weighted sample	65.4%	34.6%	63.5%	36.5%	100%
Mean Body Mass Index (SD)	25.0 (2.9)	34.8 (4.7)	27.2 (5.1)	29.6 (6.5)	28.1 (5.7)
% Reporting health as excellent/very good	69.9%	51.5%	100%	0%	64.7%
High School Student Composition					
Mean percent of students classified as disadvantaged (SD)	17.9% (20.1%)	20.8% (21.7%)	17.5% (19.7%)	21.5% (22.5%)	18.8% (20.7%)
Mean percent of students who were White (SD)	80.1% (26.1%)	76.3% (28.7%)	80.3% (25.9%)	75.6% (28.7%)	78.9% (27.0%)
Individual Characteristics					
Childhood socioeconomic position					
Mean years of maternal education (SD)	11.9 (2.6)	11.4 (2.7)	12.0 (2.5)	11.2 (2.7)	11.7 (2.6)
Mean paternal education (SD)	12.1 (3.4)	11.7 (3.5)	12.4 (3.4)	11.3 (3.5)	12.0 (3.5)
Lived in an urban setting as a child	77.2%	73.8%	78.4%	73.0%	76.1%
Lived in the South as a child	31.1%	36.4%	29.8%	37.6%	32.7%
Spoke a foreign language as a child	12.1%	11.8%	12.3%	12.1%	12.0%
Demographics					
Hispanic	3.6%	5.2%	3.8%	5.2%	4.1%
Non-Hispanic Black	10.5%	16.5%	10.5%	16.3%	12.4%
Non-Black non-Hispanic	85.9%	78.3%	85.7%	78.5%	83.5%
Female	50.7%	49.5%	49.6%	51.6%	50.3%
Educational attainment at age 25					
Did not graduate from high school	10.8%	10.9%	9.1%	17.1%	10.9%
Graduated from high school but not college	64.5%	72.6%	63.4%	71.1%	67.0%
Graduated from college or beyond	24.7%	16.5%	27.5%	11.8%	22.1%

Sample size reported indicates all individuals for whom information was available on all three measures of high school student composition. For the total population column, the sample size reported is the number of individuals for whom information was available on all student composition measures and both health measures.

**Table 2:** Ordered odds ratios for self-rated health at age 40.

High School Student Composition Characteristics	Model 1	Model 2	Model 3
Percent of students classified as disadvantaged (in 5 percentage point increments)	0.96 (95% CI: 0.95, 0.97) (*n* = 5005)	0.99 (95% CI: 0.97, 1.00) (*n* = 4191)	0.99 (95% CI: 0.97, 1.01) (*n* = 4094)
Percent of students who were white (in 5 percentage point increments)	1.03 (95% CI: 1.02, 1.04) (*n* = 5802)	1.00 (95% CI: 0.99, 1.02) (*n* = 4863)	1.00 (95% CI: 0.98, 1.01) (*n* = 4757)

Model 1: bivariate association, adjusting for no confounders, Model 2: adjusts for parental (maternal and paternal) education, childhood residential geography (urbanicity, living in the south), speaking a foreign language as a child, birth year, race/ethnicity, and gender, Model 3: adjusts for variables listed in model 2 plus educational attainment at age 25. All models use sampling weights (pweights) for national representativeness.

**Table 3:** Odds ratios for obesity at age 40.

High School Student Composition Characteristics	Model 1	Model 2	Model 3
Percent of students classified as disadvantaged (in 5 percentage point increments)	1.03 (95% CI: 1.02, 1.05) (*n* = 4464)	1.01 (95% CI: 0.99, 1.03) (*n* = 3760)	1.01 (95% CI: 0.99, 1.03) (*n* = 3704)
Percent of students who were White (in 5 percentage point increments)	0.98 (95% CI: 0.97, 0.99) (*n* = 5166)	1.00 (95% CI: 0.98, 1.02) (*n* = 4355)	1.00 (95% CI: 0.98, 1.02) (*n* = 4294)

Model 1: bivariate association, adjusting for no confounders, Model 2: adjusts for parental (maternal and paternal) education, childhood residential geography (urbanicity, living in the south), speaking a foreign language as a child, birth year, race/ethnicity, and gender, Model 3: adjusts for variables listed in model 2 plus educational attainment at age 25. All models use sampling weights (pweights) for national representativeness.

## Data Availability

The data are available at: https://www.nlsinfo.org/investigator/pages/login (accessed on 30 March 2021).
